# Hemiplegia in Pneumonia of Children

**Published:** 1890-08-16

**Authors:** 


### HEMIPLEGIA IN PNEUMONIA OF CHILDREN.

Dr. Aufrecht, of Magdeburg, describes some cases in which the onset of pneumonia in young children was accompanied by coma and hemiplegia, passing off as the disease became more developed.

(1) A child aged 1¾ years was attacked with pneumonia of the upper lobe of the right lung, the disease beginning in a somewhat unusual manner, and proceeding irregularly. Coma set in on the third day, followed on the eighth by complete hemiplegia of the left side. On the ninth the mind was still confused, but became clear by night after a profuse and apparently critical perspiration, which left the child feeling better, though the paralysis remained complete. On the twelfth day some movement was observed in the hand, and later on in the foot, and by the fourteenth power was fully recovered. Sensibility had been unaffected throughout.

(2) Another child aged 2½ years was seized with fever and vomiting on May 24th, followed next day by convulsions, with left hemiplegia and constant to and fro movements of the head and neck. The paralysis passed off in a few hours, but the chronic spasms of the neck continued for some days. On May 29th he coughed for the first time, and on the 30th became unconscious, and lay perfectly apathetic. On the 31st pneumonia was clearly manifested in the upper lobe of the left lung, and consciousness returned on the next day. The child made thenceforth a good recovery.

Dr. Aufrecht, rejecting Lepine's hypothesis of cerebral ischæmia, thinks that in pneumonia, as in uræmia, coma and paralysis are induced by œdema of the brain consequent on alterations in the constitution of the blood, and that it is more frequent in pneumonia affecting the upper lobes, owing to the impediment presented thereby to the return of venous blood from the brain to the heart.

